# Effect of cognitive remediation therapy in anorexia nervosa: a systematic review and meta-analysis

**DOI:** 10.3389/fpsyt.2024.1484457

**Published:** 2024-10-30

**Authors:** Anas R. Alserihi, Wejdan A. Hubayni, Solaf Hilal Alotaibi, Sadeem Bahkali, Shatha Alqurashi, Muhannad Sadakah Abualola, Ahmad Mohammed Alsaleh

**Affiliations:** ^1^ Department of Mental Health, Johns Hopkins Aramco Healthcare, Dhahran, Saudi Arabia; ^2^ College of Medicine, King Saud bin Abdulaziz University for Health Sciences, Jeddah, Saudi Arabia; ^3^ King Abdullah International Medical Research Center, Jeddah, Saudi Arabia; ^4^ Mental Health Section, Department of Medicine, King Abdulaziz Medical City, National Guard Health Affairs, Jeddah, Saudi Arabia

**Keywords:** anorexia nervosa, an, eating disorder, cognitive remediation therapy, CRT, systematic review, meta-analysis

## Abstract

**Background:**

Anorexia nervosa (AN) can significantly affect cognitive well-being. Cognitive remediation therapy (CRT) is regarded as one of the effective treatments for cognitive impairment in some mental illnesses such as schizophrenia, bipolar disorder, and attention deficit. For this reason, this systematic review and meta-analysis aim to assess the effectiveness of CRT in patients with AN.

**Methods:**

We conducted a search of Medline, ClinicalTrials.gov, and the Cochrane Database of Systematic Reviews from the inception of each database through April 8, 2023. Randomized clinical trials evaluating the effectiveness of CRT in comparison to placebo or other psychological treatments in patients with AN were included. The quality of the studies was assessed using the revised Cochrane risk-of-bias tool. For meta-analysis, effect sizes were measured using mean difference (MD) utilizing the random-effects model and inverse variance (IV) technique. To evaluate the certainty of the evidence, we applied the Grading of Recommendations Assessment, Development, and Evaluation (GRADE) criteria. The study was registered in PROSPERO, ID: CRD42023411784.

**Results:**

In the systematic review, six studies were included, of which four underwent meta-analysis. Among these, three trials encompassing 413 participants showed that CRT was associated with improved cognitive flexibility compared to control at the end of treatment (MD = -0.21, 95% CI [-0.44, 0.02], P=0.81, I^2^ = 0%). In two trials with 143 patients, those who received CRT showed similar effects on the severity of AN symptoms compared to the control group in the self-reporting questionnaires: EDE-Q (MD = -0.25, 95% CI [-0.76, 0.27], P=0.77, I^2^ = 0%) and EDEQOL (MD = -0.19, 95% CI [-0.41, 0.03], P=0.84, I^2^ = 0%).

**Conclusion:**

CRT did not show a statistically significant difference compared to the control group in improving abstract thinking skills and quality of life in individuals with AN. That indicates that CRT’s efficacy remains inconclusive. Further research with larger, more diverse samples is needed to determine its long-term effects and potential benefits.

**Systematic review registration:**

PROSPERO, identifier CRD42023411784.

## 1 Introduction

Anorexia nervosa (AN) is a severe and complex mental health disorder characterized by persistent restriction of energy intake, an intense fear of weight gain, and a distorted self-perception regarding body weight and shape. Individuals with AN often exhibit a range of psychological symptoms, including extreme preoccupation with food, weight, and body image, which can manifest as obsessive thoughts and behaviors surrounding eating and weight control (1).. This condition is associated with significant mortality risks, as evidenced by mortality ratios indicating that the death rate in AN is approximately six times higher than in the general population (2). The psychological distress experienced by individuals with AN often leads to social withdrawal, irritability, and mood disturbances such as anxiety and depression (3, 4) Physically, AN can result in severe malnutrition, leading to a host of medical complications that affect various organ systems. Common physical symptoms include significant weight loss, bradycardia (slow heart rate), hypotension (low blood pressure), electrolyte imbalances, and amenorrhea (loss of menstrual periods) (5, 6). These physiological changes can have life-threatening consequences. Hence, there is a pressing demand for interventions that enhance outcomes and encourage adherence.

Evidence points to cognitive impairment as a central feature in the realm of anorexia nervosa neuroscience, hindering recovery and contributing significantly to the maintenance of the disorder. Executive functioning, which involves abilities such as decision-making, problem-solving, and impulse control, is often compromised in individuals with anorexia nervosa, leading to difficulties in making rational choices about food, body image, and overall health (7). Consequently, there has been growing interest in the use of cognitive remediation therapy (CRT) as a complementary approach to the treatment of anorexia nervosa. CRT is a promising adjunctive treatment, targeting cognitive deficits such as impaired set-shifting and weak central coherence. CRT aims to improve cognitive flexibility and executive functioning, which are often compromised in individuals with AN (1). Studies have demonstrated that CRT can enhance cognitive performance and facilitate engagement in more intensive psychological therapies. Moreover, CRT’s acceptability and feasibility have been confirmed across various formats, including individual, group, and family-based interventions. Preliminary findings indicate that CRT can yield significant improvements in cognitive processing and may help reduce treatment drop-out rates, thereby improving overall treatment outcomes for AN patient (8).

The outcome of a previous case report conducted in Berlin about a 12-year-old with extraordinarily severe and chronic AN concluded that CRT may be a promising add-on therapy in the clinical treatment of young girls with AN (9). Moreover, a feasibility study included twenty-two (12–18 years) participants who concluded after completing four CRT sessions that CRT appears to be associated with minor to medium effect size improvements in set-shifting (SS) and central coherence (CC). The results of this study cannot be generalized to patients with mild or severe AN presentation as the participants’ illness duration was within three years only (10).

However, results have been less favorable in a large randomized controlled trial. After examining the clinical outcomes of CRT in 167 adults and adolescents (≥17 years) with AN, the study suggested that CRT has limited short-term benefits regarding improvements in neurocognition. That is, CRT did not lead to more significant improvements in the participants eating disorder psychopathology, BMI, and health-related QoL from baseline to 6-month follow-up. The authors concluded that CRT was not more effective than standard treatments regarding clinical outcomes (11). Another randomized controlled trial studied the effects of CRT on neurocognitive performance in fifty-sex female adolescents (age = 11–17 years) with AN revealed no superiority of CRT over non-specific cognitive training at the end of their treatment (12). Therefore, there is a great need for more randomized control trials (RCTs) in different age groups with AN to overcome the limitations of the previous studies. Since then, many updated RCTs have been introduced to the literature comparing the intervention of CRT patients with AV with other treatment methods. This review aims to assess the effectiveness of CRT in patients with AN by incorporating updated RCTs and utilizing meta-analytic techniques. Specifically, we focus on evaluating both cognitive and psychological outcomes, including cognitive flexibility, set-shifting abilities, and quality of life, which have not been extensively covered in previous reviews.

## 2 Materials and methods

### 2.1 Headings data sources and search strategy

This study followed the Preferred Reporting Items for Systematic Reviews and Meta-Analyses (PRISMA) guidelines (13). Prior to conducting the initial search, a protocol was formulated and officially registered with PROSPERO (CRD42023411784). We conducted a comprehensive search across Medline, ClinicalTrials.gov, and the Cochrane Database of Systematic Reviews from the inception of each database through April 8, 2023. The search terms included a combination of keywords related to ‘Anorexia Nervosa,’ ‘Cognitive Remediation Therapy,’ and ‘Randomized Controlled Trials.’ Boolean operators (AND, OR) were used to refine the search. Specific search keys tailored to each database were utilized, and filters were applied to include only randomized controlled trials (RCTs) published in English. Studies in other languages were excluded to ensure consistent interpretation of the findings. A detailed description of the complete search strategy, including specific queries for each database, is provided in the **Supplementary Material**.

### 2.2 Study selection

In this systematic review, we applied the PICOS framework (Population, Intervention, Comparison, Outcome, and Study design). The included studies focused on patients with anorexia nervosa (Population) who underwent Cognitive Remediation Therapy (Intervention). The comparison group (Comparison) comprised patients receiving standard care or other psychological treatment. The primary outcomes (Outcome) assessed were improvements in abstract thinking skills and quality of life. The study designs considered were RCTs. The exclusion criteria were (1) single-arm randomized clinical trials, (2) observational studies, case reports, and RCT protocols. All included studies were evaluated by two independent reviewers. Six authors were grouped into three pairs (ARA and WAH, SHA and SA, SB and MSA). The assessment was independently and initially based on the title and abstract, followed by reviewing the full text of the articles. If there was a disagreement between the two reviewers, a third reviewer was contacted to conduct an independent review from an expert (AA). Eventually, only studies that met the previously defined criteria were selected.

### 2.3 Data extraction and risk of bias assessment

Two independent investigators extracted the data. The following data was extracted from each eligible study: first author’s name, county, publication year, number of participants of intervention, number of participants of comparison, participants demographics (sex and mean age), intervention setting (inpatient or outpatient), mode of intervention delivery (individual or group), number of intervention session, intervention intensity (frequency and duration), outcome measures. The quality of evidence of the included studies was evaluated by two independent authors using the Revised Cochrane risk-of-bias tool for randomized trials (RoB 2) (14). According to their assessment, each study was categorized as high, low, or with some concerns. Conflicts were resolved either via consensus or by contacting a third author.

### 2.4 Outcomes

The study measured effect sizes for different outcomes to evaluate the effectiveness of CRT in patients with AN. The Wisconsin Card Sorting Test (WCST) was used to evaluate abstract reasoning skills and the capacity to adapt cognitive strategies to assess AN severity; the Eating Disorder Examination Questionnaire (EDE-Q) was used. The Eating Disorder Quality of Life Scale (EDQOL) was used to assess the quality of life among patients with eating disorders. Mean and standard deviation (SD) values were derived from the study data collected at baseline and after treatment.

### 2.5 Statistical analysis and quality assessment

The data was analyzed using RevMan (Review Manager) version 5.4 (Cochrane Collaboration). A random-effects model was used, and all data were pooled utilizing the inverse variance (IV) weighting technique. The statistical significance was established at a p-value of less than 0.05. Mean Difference (MD) with their 95% confidence interval (CIs) calculated for continuous variables. The statistical heterogeneity was evaluated using I^2^ statistics, where I^2^ values exceeding 50% indicated significant heterogeneity. When there were multiple observations of outcomes, the most recent follow-up visit was pooled in the meta-analysis. The certainty of the evidence of each estimate was evaluated utilizing the Grading of Recommendations, Assessment, Development, and Evaluation (GRADE) assessment tool by assessing the study design, the quantity of included studies, bias risk, inconsistency, indirectness, and imprecision (15). Due to the limited number of included studies, publication bias could not be assessed utilizing funnel plots or other assessment methods such as Egger’s test (16). After extracting the data, **Table 1** was generated to display the information.

**Table 1:** Trail characteristics.

Author	Country	Number of participants	Sex	Mean age	Intervention				Control	Outcomes
					Setting	Mode of delivery	quantity	Intensity		
Sproch et al., 2019 (17)	USA	Intervention: 135; Control: 140	Male: 25; Female: 250	CRT: 23.9 (12.8); TAU: 22.2 (12.8)	In-patient	Group	5	Twice a week and then once a week	Treatment as usual (TAU)	WCST; CTMT; CBT
van Passel et al., 2020 (18)	The Netherlands	Intervention: 31; Control: 30	Male: 4; Female: 57	CRT: 25.19 (7.66); SAT: 24.60 (6.98)	Out-patient	Individual	10	Twice-weekly 45-min sessions	SAT + TAU	EDE-Q; EDQOL; DFlex
Timko et al., 2023 (19)	USA	Intervention: Parent: 57, adolescent: 57; Control: 63	Male: 67; Female: 110	Adolescents: 15.335 (1.641); mothers: 47.66 (4.42); fathers: 49.88 (5.87)	Out-patient	Individual /Family	15	Fifteen sessions of Treatment over six months.	Family Based Treatment (FBT)	D-KEFS; BRIEF
Herbrich‐Bowe et al., 2022 (20)	Germany	Intervention: 28; Control: 28	Male: 0; Female: 56	CRT: 14.8 (1.5); NSCT: 15.3 (1.6)	In-patient	Individual	10	Twice weekly over 5 weeks	NSCT	WSCT; TMT‐4; GEFT; BRIEF
Brockmeyer et al., 2021 (8)	Germany	Intervention: 82; Control: 85	Male: 4; Female: 163	CRT: 26.06 (9.06), ART: 25.58 (8.62)	In-patient	Group	10	One session per week for 10 weeks	ART	BMI; EDE-Q; ELI; SOCQED; DFlex; PNS; IUS; WCST; TMT; NT
Dingemans et al., 2013 (21)	The Netherlands	Intervention: 41; Control: 41	Male: 0; Female: 82	NA	In-patient	Individual	10	45 minutes for each session Within 6 weeks	TAU	EDE-Q; EDQOL; BMI; BDI; RSE; SF-36; STAI; MPS; TMT; RCFT

WCST, Wisconsin card sorting test; CTMT, The comprehensive trail making test; EDE-Q, Eating Disorder Examination Questionnaire; EDQOL, The Eating Disorders Quality of Life questionnaire; DFlex, Detail and Flexibility questionnaire; D-KEFS, Delis Kaplan Executive Functioning System; BRIEF, Behavior Rating Inventory of Executive Functioning; TMT, Trail Making Test; GEFT, Group Embedded Figures Test; ELI, Essen QoL Index for Eating Disorders; SOCQ-ED, Stages of Change Questionnaire for Eating Disorders; PNS, Personal Need for Structure Scale; IUS, Intolerance of Uncertainty Scale; NT, Navon Task; BDI, Beck Depression Inventory-II; RSE, Rosenberg’s Self-Esteem Scale; SF-36 = 36-item Medical Outcomes Study Short- Form Health Survey for QoL 2; STAI, State-Trait Anxiety Inventory for anxiety; MPS, Multidimensional Perfectionism Scale for level of perfectionism; RCFT, Rey Complex Figure Task for central coherence.

## 3 Results

**Figure 1:**
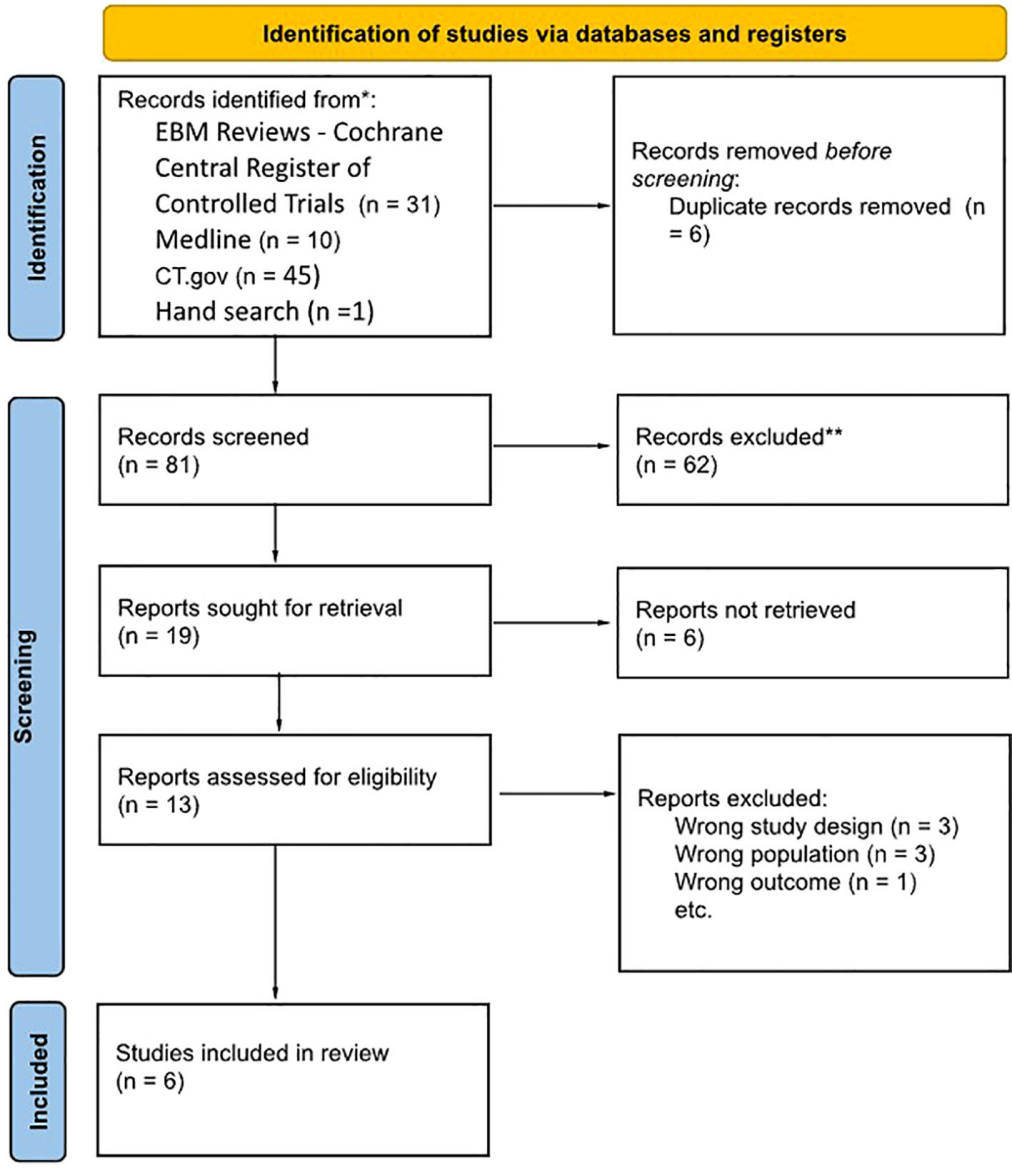
A flowchart depicting the study procedure in accordance with the guidelines outlined in the Preferred Reporting Items for Systematic Reviews and Meta-Analysis (PRISMA. Medline, CT.gov, and Cochrane. The search results were compiled in April 2023. Exclusions were carried out exclusively by human reviewers.

The systematic search initially yielded 87 reports, all screened after deduplication. Nineteen reports were considered for retrieval. Following a full-text assessment, seven studies were excluded for various reasons, such as incorrect study design, population, or outcome, while six were included (refer to **Figure 1**).

### 2.2 Trial characteristics

All six studies investigated the effect of CRT on the treatment of AN. However, the control group or the other arms were different in each study. Two of them had a control group that received treatment as usual (TAU) (17, 21). Three studies had control groups with specific therapy other than TAU, including art therapy, specialized attention therapy, and non-specific cognitive therapy (8, 18, 20). Another study assessed family behavioral therapy (FBT) alone, FBT with parent-focused CRT, and FBT with adolescent-focused CRT (22). (**Table 1**).

### 2.3 Risk of bias assessment

**Figure 2:**
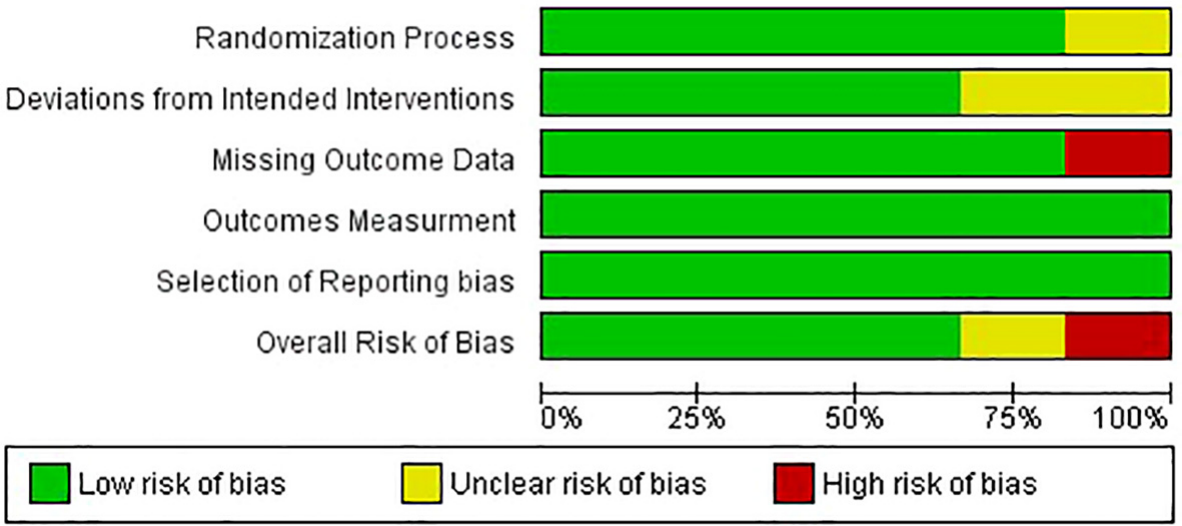
Risk of bias graph.

**Figure 3:**
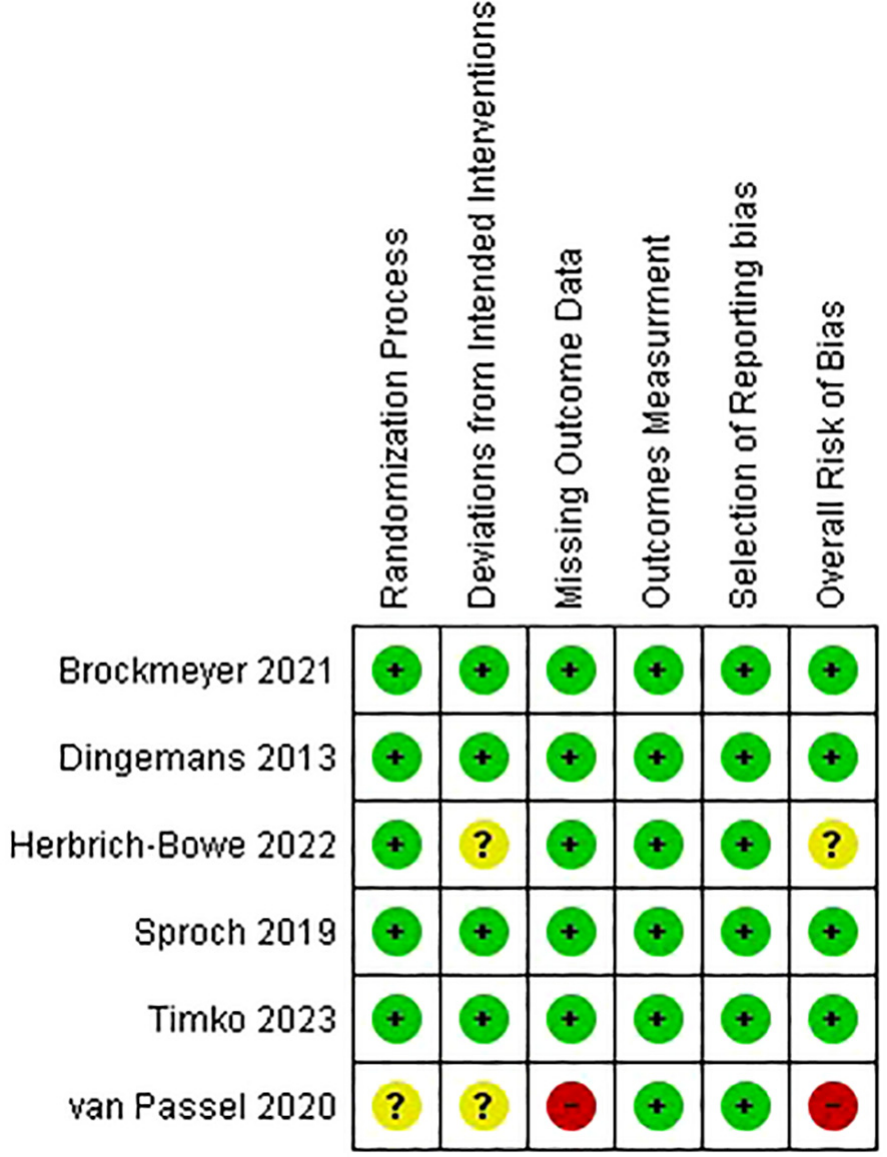
Risk of bias summary.

Four papers were categorized as low risk, while the remaining two underwent assessment, one being flagged for some concerns and the other being deemed at high risk (**Figures 2**, **3**).

### 2.4 Qualitative synthesis

Sproch et al. aimed to evaluate the effect of brief, CRT for AN onset-shifting. There was no difference between all measures of the WCST regarding treatment effect; however, there was a statistically significant time effect (17). Dingemans et al. investigated the effectiveness of CRT plus TAU for patients with a severe or enduring eating disorder in comparison to TAU alone. Similar to what we found in the analysis; their results showed significant improvement in the experimental group who received CRT in addition to TAU (21). Herbrich‐Bowe et al. compared CRT to non-specific cognitive training (NSCT) in adolescent inpatients with AN. They found that both groups improved over time, but NSCT was superior to CRT in terms of self‐reported planning/organization ability. Dingemans et al. also used a global mean scale of EDE-Q and EDQOL to measure their outcomes. The first scale was used to measure psychopathology, and the second was used for quality-of-life assessment. They found that both groups showed improvement regarding psychopathology; the CRT group showed more significant improvement than the control group in both scales (20). Passel et al. studied the effect of CRT compared to specialized attention therapy (SAT) and both as add-ons. They used the same scales used by Dingemans et al. to measure their outcomes except WCST. Contrary to what they expected, SAT was more effective in patients with obsessive-compulsive disorder (OCD). Other than that, CRT was not superior to SAT (18). Regarding cognitive and clinical outcomes, Brockmeyer et al. studied the effectiveness as an add-on to TAU. Their results stated that CRT was similar to TAU in all aspects except for more significant short-term improvements in one measure of set-shifting (8).

### 2.5 Meta-analysis

#### 2.5.1 The Wisconsin card sorting test: number of perseverative errors

**Figure 4:**
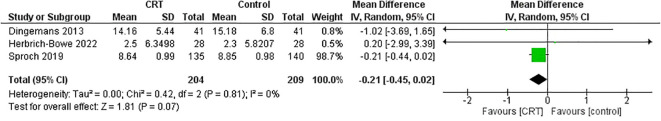
Forest plot of WSCR error. CI, confidence interval; IV, inverse variance; RR, risk ratio.

**Figure 5:**
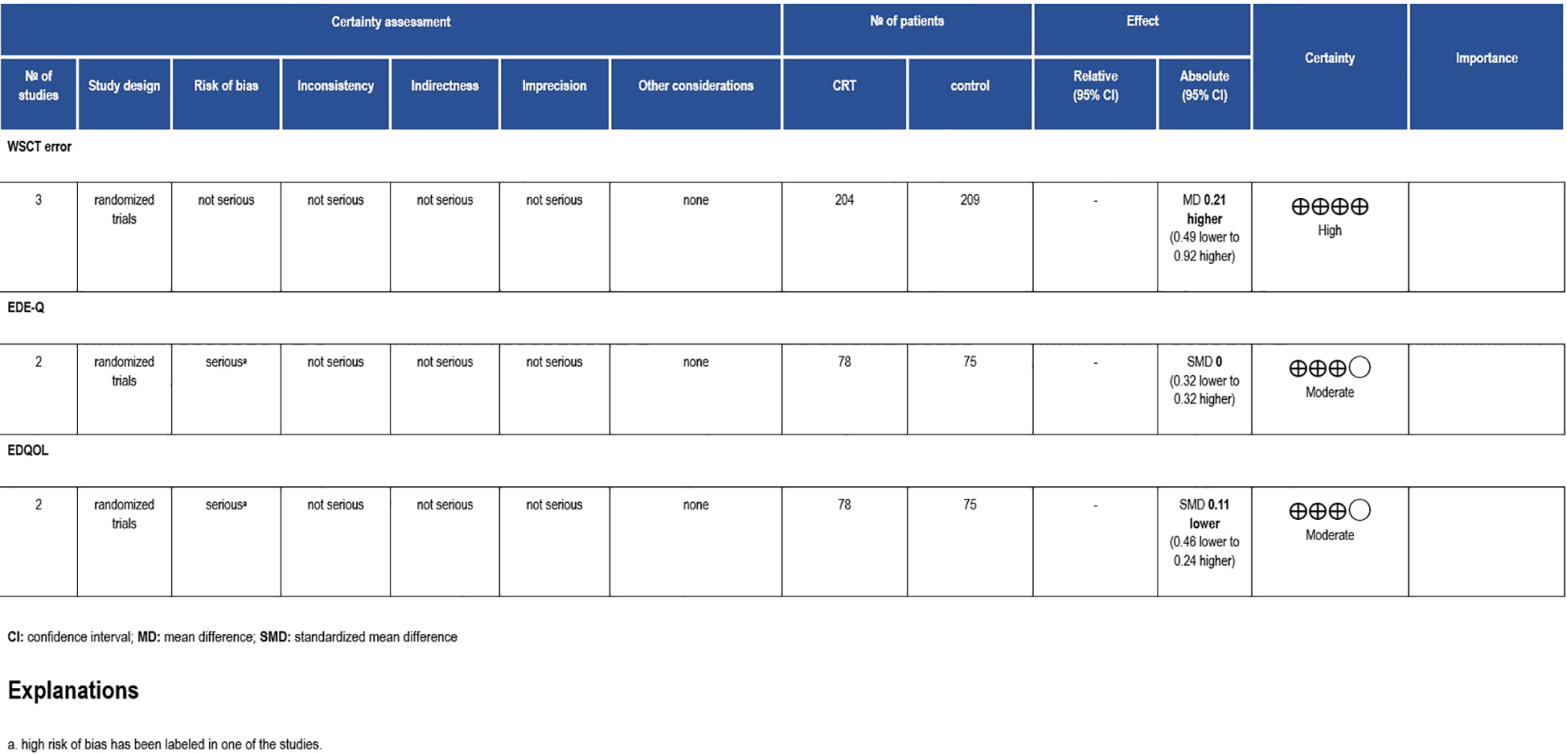
Grading of Recommendations Assessment, Development and Evaluation (GRADE) evidence profile. CI, confidence interval; RCT, randomized controlled trial; RR, risk ratio.

Three trials used WCST to measure their outcomes, with 413 participants (17, 20, 21). The results indicated that the CRT group had a slightly superior effect to the control group without statistical significance (MD = -0.21, 95% CI [-0.44, 0.02], P=0.81, I2 = 0%) (**Figure 4**). High certainty of evidence was estimated in the GRADE assessment (**Figure 5**).

#### 2.5.2 Eating disorder examination questionnaire and eating disorder quality of life scale

**Figure 6:**
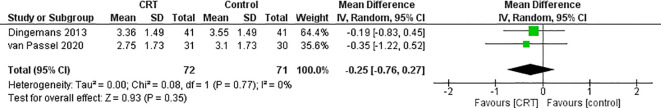
Forest plot of EDE-Q. CI, confidence interval; IV, inverse variance; RR, risk ratio.

**Figure 7:**
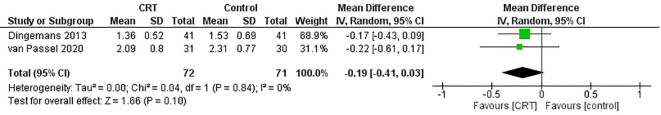
Forest plot of EDQOL. CI, confidence interval; IV, inverse variance; RR, risk ratio.

With approximately 143 patients, two studies reported their results using EDE-Q and EDEQOL scales (18, 21). EDE-Q showed that the experimental group who received CRT had a homogeneous effect to the control group (MD = -0.25, 95% CI [-0.76, 0.27], P=0.77, I2 = 0%) (**Figures 6**, **7**), and this is labeled as moderate certainty of evidence (**Figure 5**). Similarly, the same results were reported by EDEQOL (MD = -0.19, 95% CI [-0.41, 0.03], P=0.84, I2 = 0%) with moderate certainty of evidence (**Figure 5**).

## 3 Discussion

AN is an eating disorder characterized by extreme concerns about weight and shape, leading to restrictive or purging eating behaviors and significant weight loss. It involves a complex interplay of biological, psychological, and sociocultural factors. Individuals with this disorder experience an intense fear of gaining weight, a distorted body image, and engage in restrictive eating behaviors. The resulting malnutrition and weight loss can cause severe medical complications, affecting various organ systems. Additionally, AN has the highest mortality rate among psychiatric disorders (2, 2). The standard treatment for AN generally involves nutritional rehabilitation and psychotherapy (23). Although several therapies have been developed or adapted to address this disorder, there is no compelling empirical evidence demonstrating the clear superiority of one therapy over another in terms of effectiveness (24).

However, CRT has shown promise as an effective adjunctive treatment for AN and other eating disorders by targeting cognitive deficits that contribute to the persistence of these conditions (25, 26). Research indicates that CRT can enhance cognitive flexibility, reduce rigid thinking, and improve overall treatment engagement among patients (25, 27). A longitudinal study demonstrated significant improvements in clinical symptoms and cognitive styles among AN patients participating in a rolling group CRT intervention within an inpatient setting (28). These findings suggest that CRT is feasible and beneficial for both restrictive and binge-purge subtypes of AN, highlighting its potential to facilitate recovery by addressing the neuropsychological profiles associated with eating disorders. As such, integrating CRT into comprehensive treatment plans may enhance therapeutic outcomes and support sustained recovery efforts.

Since the last systematic review examining the efficacy of CRT in treating AN, several new RCTs have been conducted (12). These recent studies have provided valuable insights into the effectiveness of CRT, particularly in the context of AN and severe or enduring eating disorders. In light of these developments, we conducted this updated systematic review and meta-analysis to synthesize the latest evidence and provide a comprehensive overview of the impact of CRT on cognitive functions, psychopathology, and quality of life.

Our analysis, which encompassed several studies investigating CRT for individuals with AN, found limited improvements in cognitive flexibility and psychological outcomes. For example, Sproch et al. found no significant treatment effects on set-shifting abilities using the WCST, while Dingemans et al. reported significant improvements in psychopathology and quality of life when CRT was augmented with TAU (17, 21). In contrast, Herbrich-Bowe et al. favored NSCT over CRT in improving self-reported planning abilities (20). However, Brockmeyer et al. concluded that CRT, when combined with TAU, yielded similar results to control treatments, with some short-term improvements in set-shifting observed in the CRT group (8). Overall, the meta-analysis across these studies indicated no significant differences between CRT and control groups.

Our findings closely align with those of Hagan et al., who also reported no statistically significant improvements in cognitive flexibility, set-shifting abilities, or quality of life in AN patients undergoing CRT (12). While both studies noted variability in set-shifting measures, our review expanded upon their work by including additional tools beyond the WCST to assess cognitive flexibility. Like Hagan et al., we found no significant advantage of CRT over control treatments in reducing eating disorder symptoms or enhancing psychological outcomes.

Moreover, a recent systematic review of systematic reviews and meta-analyses has been recently published (29). However, notable differences arise in several key aspects. Firstly, and most importantly, there is a significant difference in the inclusion criteria. Our study specifically focused on randomized clinical trials compared to the recent systematic review, which included systematic reviews. This disparity in inclusion criteria is particularly significant as recent randomized clinical trials have been conducted. Our systematic review is the first attempt to summarize and analyze their findings. Secondly, our systematic review involved conducting a meta-analysis to analyze the data, leading to a more comprehensive understanding of cognitive remediation therapy’s efficacy in treating AN. In contrast, their review relied solely on a qualitative summary of the findings. We note that no additional RCTs were included in their studies beyond our search period, which were not already part of our current systematic review.

Additionally, recent developments in cognitive remediation for AN have led to the introduction of Cognitive Remediation and Emotion Skills Training (CREST), which integrates emotional skills training with traditional cognitive remediation therapy. This represents an advancement over the standard CRT protocol, as it addresses both cognitive flexibility and emotional regulation deficits, which are common in AN. Preliminary studies on CREST suggest that it may offer additional benefits in improving emotional processing alongside cognitive skills, making it a promising approach for future interventions (30). Incorporating CREST into future research may enhance the effectiveness of CRT by addressing the emotional challenges that contribute to treatment resistance in AN.

### 3.1 Limitations

One notable limitation of this systematic review is the language restriction applied during the search process. Our study was limited to articles and data in English, potentially excluding relevant studies published in other languages. Additionally, our search strategy was focused on specific databases, including Medline, ClinicalTrials.gov, and the Cochrane Database of Systematic Reviews, which may have resulted in the omission of studies indexed in other databases or grey literature sources. We also did not extract data on ethnic diversity, which limits the applicability of our results to diverse populations. Future studies should consider including more languages, broader databases, and explicitly reports on ethnic diversity to improve the generalizability of findings.

### 3.2 Conclusion

Based on the findings, it can be concluded that while CRT shows potential in some cognitive and clinical aspects, it does not demonstrate a statistically significant difference compared to the control group for individuals with AN. That indicates that the efficacy of CRT in treating such conditions remains inconclusive based on the current data. Therefore, further randomized controlled studies with larger sample sizes and more extended follow-up periods are needed to clarify the long-term effects and potential benefits of CRT for individuals with anorexia nervosa.

## Data Availability

The original contributions presented in the study are included in the article/**Supplementary Material**, further inquiries can be directed to the corresponding author/s.
