# Supplementary material for: Effect of cognitive remediation therapy in anorexia nervosa: a systematic review and meta-analysis
Source: Front Psychiatry. 2024 Oct 30;15:1484457. doi: 10.3389/fpsyt.2024.1484457 (PMC11557350; doi:10.3389/fpsyt.2024.1484457)
Supplement: Supplementary file 1 [file Table1.docx]

Supplementary Material

# Search Strategy

EBM Reviews - Cochrane Central Register of Controlled Trials <March 2023>

1 exp Anorexia Nervosa/ 712

2 anorexia nervosa$.mp. 1293

3 1 or 2 1293

4 Cognitive Remediation Therapy$.mp. 501

5 Cognitive Enhancement Therapy$.mp. 60

6 Cognitive Rehabilitation Therapy$.mp. 49

7 4 or 5 or 6 591

8 exp Randomized Controlled Trial/ or exp Clinical Trial/ or exp Controlled Clinical Trial/ 45358

9 trial$.mp. 1088291

10 8 or 9 1088291

11 3 and 7 and 10 31

Total: 31

Medline search strategy (8/4/2023)

| Search | Query | Results |
| --- | --- | --- |
| #4 | Search:  #1 AND #2 AND #3 | 10 |
| #3 | Search:  **(((controlled clinical trials, randomized[MeSH Terms]) OR (clinical trial[MeSH Terms])) OR (randomized controlled trial[MeSH Terms])) OR (clinical trials, randomized[MeSH Terms])** | 381,368 |
| #2 | Search:  **(((Cognitive Remediation Therapy[MeSH Terms]) OR (CRT[MeSH Terms])) OR (Cognitive Remediation Therapy[Title/Abstract])) OR (CRT[Title/Abstract])** | 20,370 |
| #1 | Search:  **((((((((((anorexia[MeSH Terms]) OR (anorexia nervosa[MeSH Terms])) OR (anorexia nervosas[MeSH Terms])) OR (anorexias[MeSH Terms])) OR (nervosa, anorexia[MeSH Terms])) OR (AN[MeSH Terms])) OR (anorexia[Title/Abstract])) OR (Anorexia nervosa[Title/Abstract])) OR (anorexia nervosas[Title/Abstract])) OR (anorexias[Title/Abstract])) OR (nervosa, anorexia[Title/Abstract])** | 38,704 |

Total: 10

CT.gov search strategy: (8/4/2023)

Condition: Anorexia Nervosa OR Anorexia OR AN

Other terms: Cognitive Remediation Therapy OR CRT OR Cognitive Rehabilitation Therapy OR Cognitive Enhancement Therapy

Search Limits: Study Type [interventional] Study results [with results]

Total: 45
